# Three cases report of resistance to thyroid hormone and the genetic mutation of these patients and their family members

**DOI:** 10.1186/1687-9856-2013-S1-P155

**Published:** 2013-10-03

**Authors:** Yi Gu, Chun-xiu Gong, Qian Dong, Chang Su

**Affiliations:** 1 The Capital medical University, Beijing Children’s Hospital, Beijing 100045, China

## Aims

We report the clinical characteristics and the genetic analysis of their family members of 3 cases. Resistance to thyroid hormone (RTH), a dominant inherited syndrome and it is usually due to mutations located at the ligand-binding domain and adjacent hinge region of the thyroid hormone receptor β (TRβ).

## Methods

We describe patients’ clinical features, biochemical and hormones level such as thyroid function tests (TFTs), imagination data. We also detected the TFTs of his family members as well. Direct DNA sequencing of the TRβ gene was done for all those with abnormal TFTs.

## Results

The RTH children had goiter, irritability, aggressiveness, and hyperhidrosis. The TFTs showed high levels of circulating free thyroid hormones (FT4 and FT3) and normal or high thyroid-stimulating hormone (TSH) concentrations. All of these patients used bromocriptine, and then clinical presentations got improved obviously without obvious advert effect. From these three patients and their family members we identified a novel missense mutation, A317D, located in exon 9 of the gene of a boy patient and his mother, but his mother showed no any clinical presentation. However, we didn't have any abnormal finding in for other patients and their parents.

## Conclusion

The RTH children had goiter, irritability, aggressiveness, and hyperhidrosis. The TFTs showed high levels of circulating free thyroid hormones (FT4 and FT3) and normal or high thyroid-stimulating hormone (TSH) concentrations. Fund a novel mutation in the TRβ in one patient and his mother. This research verified the phenomena that there is a heterogeneous within the same mutation of RTH patients. All of these patients used bromocriptine, and then clinical presentations got improved obviously without obvious advert effect.

